# Supplementary material for: A New Microporous Lanthanide Metal–Organic Framework with a Wide Range of pH Linear Response
Source: Molecules. 2022 Dec 8;27(24):8696. doi: 10.3390/molecules27248696 (PMC9780847; doi:10.3390/molecules27248696)
Supplement: Supplementary file 1 [file molecules-27-08696-s001.zip › molecules-2009515-supplementary.pdf]

Supporting Information

A New Microporous Lanthanide Metal-Organic Framework with a Wide Range of pH Linear Response

Ruyi Zhang [1], Liangliang Zhu [2,*] and Bingbing Yue [1,2,*]

[1] School of Materials and Chemistry, Shanghai Collaborative Innovation Center of Energy Therapy for Tumors, University of Shanghai for Science and Technology, Shanghai 200093, China

[2] State Key Laboratory of Molecular Engineering of Polymers, Department of Macromolecular Science, Fudan University, Shanghai 200438, China

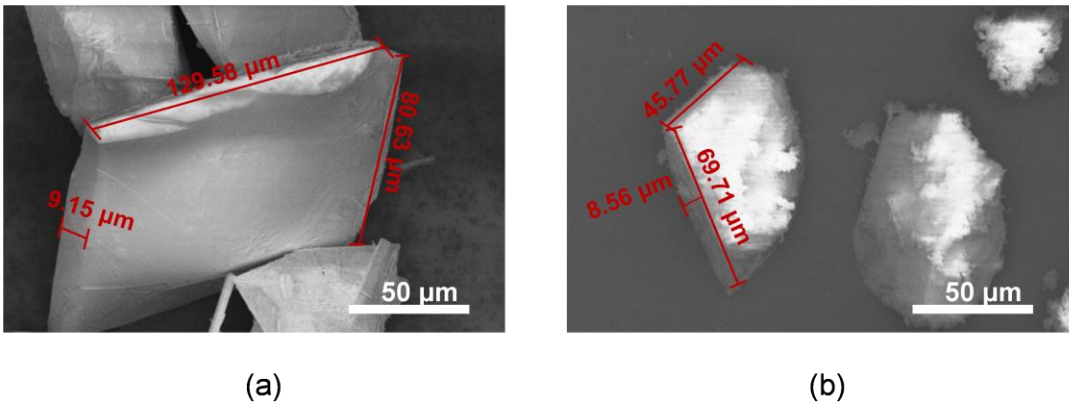

Figure S1. SEM image of as-prepared Eu-TATB (a) and Tb-TATB (b) crystal.

Table S1. Crystal data and structure refinement for Eu-TATB.

**Compound**	**Eu-TATB**	
CCDC No.	2217468	
Empirical formula	C24.30 H12.45 Eu N3 Na0.14 O7	
Formula weight	613.45	
Temperature	173(2) K	
Wavelength	1.34139 Å	
Crystal system	Orthorhombic	
Space group	Fddd	
Unit cell dimensions	a = 22.2936(9) Å	a= 90°
	b = 28.6236(17) Å	b= 90°.
	c = 28.8472(11) Å	g = 90°.
Volume	18408.1(15) Å^3	
Z	32	
Density (calculated)	1.771 Mg/m^3	

Absorption coefficient	14.525 mm^{-1}
F(000)	9591
Crystal size	0.140 x 0.100 x 0.040 mm^3
Theta range for data collection	4.361 to 63.993°.
Index ranges	-29<=h<=29, -38<=k<=38, -38<=l<=38
Reflections collected	42380
Independent reflections	5812 [R(int) = 0.0504]
Completeness to theta = 53.594°	100.00%
Absorption correction	Semi-empirical from equivalents
Max. and min. transmission	0.419 and 0.162
Refinement method	Full-matrix least-squares on F^2
Data / restraints / parameters	5812 / 26 / 335
Goodness-of-fit on F^2	1.13
Final R indices [I>2sigma(I)]	R1 = 0.0402, wR2 = 0.0992
R indices (all data)	R1 = 0.0428, wR2 = 0.1007
Extinction coefficient	n/a
Largest diff. peak and hole	1.690 and -1.787 e.$Å^{-3}$

Table S2. Bond lengths [Å] for Eu-TATB.

**Chemical bond**	**Lengths [Å]**	**Chemical bond**	**Lengths [Å]**
Eu(1)-O(6)	2.292(3)	Eu(1)-O(7')	2.466(14)
Eu(1)-O(1)#1	2.331(3)	Eu(1)-O(4)#5	2.738(4)
Eu(1)-O(5)#3	2.351(4)	O(3)-Na(1)#7	2.230(9)
Eu(1)-O(4)#4	2.376(3)	O(5)-Na(1)#3	2.600(5)
Eu(1)-O(3)#5	2.402(3)	Na(1)-O(7)	2.299(12)
Eu(1)-O(7)	2.436(7)	Na(1)-O(7)#9	2.299(12)

Table S3. Bond and angles [°] for Eu-TATB.

**Chemical bond**	**Angles [°]**	**Chemical bond**	**Angles [°]**
O(6)-Eu(1)-O(1)#1	115.17(15)	O(3)#14-Na(1)-O(3)#5	92.3(4)
O(6)-Eu(1)-O(2)#2	89.97(14)	O(3)#14-Na(1)-O(7)	170.3(3)
O(1)#1-Eu(1)-O(2)#2	134.92(14)	O(4)#4-Eu(1)-O(7')	154.1(5)
O(6)-Eu(1)-O(5)#3	83.20(13)	O(3)#5-Eu(1)-O(7')	66.6(5)
O(1)#1-Eu(1)-O(5)#3	74.77(14)	O(6)-Eu(1)-O(4)#5	150.79(12)

O(2)#2-Eu(1)-O(5)#3	148.46(15)	O(1)#1-Eu(1)-O(4)#5	73.84(13)
O(6)-Eu(1)-O(4)#4	80.62(13)	O(2)#2-Eu(1)-O(4)#5	67.78(13)
O(1)#1-Eu(1)-O(4)#4	71.92(14)	O(5)#3-Eu(1)-O(4)#5	125.66(12)
O(2)#2-Eu(1)-O(4)#4	76.59(14)	O(4)#4-Eu(1)-O(4)#5	76.07(11)
O(5)#3-Eu(1)-O(4)#4	131.82(14)	O(3)#5-Eu(1)-O(4)#5	50.33(11)
O(6)-Eu(1)-O(3)#5	151.00(14)	O(7)-Eu(1)-O(4)#5	114.4(3)
O(1)#1-Eu(1)-O(3)#5	86.77(15)	O(3)#5-Na(1)-O(7)	83.8(3)
O(2)#2-Eu(1)-O(3)#5	86.53(14)	O(3)#14-Na(1)-O(7)#9	83.8(3)
O(5)#3-Eu(1)-O(3)#5	84.87(13)	O(3)#5-Na(1)-O(7)#9	170.3(3)
O(4)#4-Eu(1)-O(3)#5	126.13(12)	O(7)-Na(1)-O(7)#9	101.5(7)
O(6)-Eu(1)-O(7)	74.0(3)	O(3)#14-Na(1)-O(5)#3	115.4(2)
O(1)#1-Eu(1)-O(7)	147.1(2)	O(3)#5-Na(1)-O(5)#3	82.85(15)
O(2)#2-Eu(1)-O(7)	73.3(3)	O(7)-Na(1)-O(5)#3	73.0(3)
O(5)#3-Eu(1)-O(7)	75.3(3)	O(7)#9-Na(1)-O(5)#3	90.8(3)
O(4)#4-Eu(1)-O(7)	140.2(2)	O(3)#14-Na(1)-O(5)#8	82.85(15)
O(3)#5-Eu(1)-O(7)	77.4(3)	O(3)#5-Na(1)-O(5)#8	115.4(2)
O(6)-Eu(1)-O(7')	84.4(5)	O(7)-Na(1)-O(5)#8	90.8(3)
O(1)#1-Eu(1)-O(7')	133.9(5)	O(7)#9-Na(1)-O(5)#8	73.0(3)
O(2)#2-Eu(1)-O(7')	82.4(5)	O(5)#3-Na(1)-O(5)#8	154.6(5)
O(5)#3-Eu(1)-O(7')	66.4(5)		

Symmetry transformations used to generate equivalent atoms:

#1 x-1/2,-y+3/4,-z+1/4 #2 -x+1,y-1/4,z-1/4 #3 -x+1/4,y,-z+1/4

#4 x-1/4,y+1/4,-z+1/2 #5 -x+3/4,-y+1/4,z-1/2

#6 -x+1/2,-y+1/2,-z #7 x+1/2,y,z+1/2 #8 x,-y+1/4,-z+1/4

#9 -x+1/4,-y+1/4,z #10 x+1/2,-y+3/4,-z+1/4 #11 -x+1,y+1/4,z+1/4

#12 -x+3/4,-y+1/4,z+1/2 #13 x+1/4,y-1/4,-z+1/2

#14 x-1/2,y,z-1/2

Table S4. Crystal data and structure refinement for Tb-TATB.

**Compound**	**Tb-TATB**
CCDC No.	2217637
Empirical formula	C48.59 H24.91 N6 Na0.27 O14 Tb2
Formula weight	1240.78
Temperature	173(2) K
Wavelength	1.34138 Å
Crystal system	Orthorhombic

Space group	Fddd
Unit cell dimensions	a = 22.2158(8) Å a= 90°.
	b = 28.5272(10) Å b= 90°.
	c = 28.6939(10) Å g = 90°.
Volume	18184.9(11) Å^3
Z	16
Density (calculated)	1.813 Mg/m^3
Absorption coefficient	16.452 mm^{-1}
F(000)	9655
Crystal size	0.160 x 0.110 x 0.030 mm^3
Theta range for data collection	3.802 to 56.499°.
Index ranges	-27<=h<=27, -35<=k<=35, -35<=l<=35
Reflections collected	56045
Independent reflections	4593 [R(int) = 0.0892]
Completeness to theta = 53.594°	100.00%
Absorption correction	Semi-empirical from equivalents
Max. and min. transmission	0.751 and 0.372
Refinement method	Full-matrix least-squares on F^2
Data / restraints / parameters	4593 / 26 / 335
Goodness-of-fit on F^2	1.094
Final R indices [I>2sigma(I)]	R1 = 0.0428, wR2 = 0.1070
R indices (all data)	R1 = 0.0536, wR2 = 0.1130
Extinction coefficient	n/a
Largest diff. peak and hole	0.725 and -1.547 e.Å^{-3}

Table S5. Bond lengths [Å] for Tb-TATB.

**Chemical bond**	**Lengths [Å]**	**Chemical bond**	**Lengths [Å]**
Tb(1)-O(6)	2.265(4)	Tb(1)-O(7)	2.430(8)
Tb(1)-O(1)#1	2.300(5)	Tb(1)-O(4)#5	2.743(5)
Tb(1)-O(2)#2	2.311(4)	O(3)-Na(1)#7	2.226(11)
Tb(1)-O(5)#3	2.316(4)	O(5)-Na(1)#3	2.610(6)
Tb(1)-O(4)#4	2.343(4)	Na(1)-O(7)#9	2.274(14)
Tb(1)-O(3)#5	2.362(4)	Na(1)-O(7)	2.274(14)
Tb(1)-O(7')	2.406(18)		

Table S6. Bond angles [°] for Tb-TATB.

Chemical bond	Angles [°]	Chemical bond	Angles [°]
O(6)-Tb(1)-O(1)#1	115.25(19)	O(6)-Tb(1)-O(3)#5	151.83(18)
O(6)-Tb(1)-O(2)#2	90.22(17)	O(1)#1-Tb(1)-O(3)#5	85.72(19)
O(1)#1-Tb(1)-O(2)#2	134.68(17)	O(2)#2-Tb(1)-O(3)#5	86.93(17)
O(6)-Tb(1)-O(5)#3	82.76(16)	O(5)#3-Tb(1)-O(3)#5	85.33(17)
O(1)#1-Tb(1)-O(5)#3	74.66(18)	O(4)#4-Tb(1)-O(3)#5	126.04(16)
O(2)#2-Tb(1)-O(5)#3	148.88(19)	O(6)-Tb(1)-O(7')	85.7(6)
O(6)-Tb(1)-O(4)#4	80.17(16)	O(1)#1-Tb(1)-O(7')	132.2(6)
O(1)#1-Tb(1)-O(4)#4	72.16(17)	O(2)#2-Tb(1)-O(7')	83.6(6)
O(2)#2-Tb(1)-O(4)#4	76.61(18)	O(5)#3-Tb(1)-O(7')	65.7(6)
O(5)#3-Tb(1)-O(4)#4	131.14(18)	O(4)#4-Tb(1)-O(7')	155.6(6)
O(3)#5-Tb(1)-O(7')	66.1(6)	O(3)#14-Na(1)-O(3)#5	94.1(6)
O(6)-Tb(1)-O(7)	74.5(3)	O(3)#14-Na(1)-O(7)#9	83.9(3)
O(1)#1-Tb(1)-O(7)	146.2(3)	O(3)#5-Na(1)-O(7)#9	170.4(3)
O(2)#2-Tb(1)-O(7)	74.1(3)	O(3)#14-Na(1)-O(7)	170.4(3)
O(5)#3-Tb(1)-O(7)	74.8(3)	O(3)#5-Na(1)-O(7)	83.9(3)
O(4)#4-Tb(1)-O(7)	140.8(3)	O(7)#9-Na(1)-O(7)	99.7(8)
O(3)#5-Tb(1)-O(7)	77.8(3)	O(3)#14-Na(1)-O(5)#3	117.0(3)
O(6)-Tb(1)-O(4)#5	150.64(14)	O(3)#5-Na(1)-O(5)#3	81.50(19)
O(1)#1-Tb(1)-O(4)#5	73.25(17)	O(7)-Na(1)-O(5)#3	72.0(3)
O(2)#2-Tb(1)-O(4)#5	67.85(16)	O(3)#14-Na(1)-O(5)#8	81.50(19)
O(5)#3-Tb(1)-O(4)#5	126.09(15)	O(3)#5-Na(1)-O(5)#8	117.0(3)
O(4)#4-Tb(1)-O(4)#5	76.01(15)	O(7)#9-Na(1)-O(5)#8	72.0(3)
O(3)#5-Tb(1)-O(4)#5	50.34(14)	O(7)-Na(1)-O(5)#8	91.1(4)
O(7)-Tb(1)-O(4)#5	115.0(3)	O(5)#3-Na(1)-O(5)#8	154.1(7)
O(7)#9-Na(1)-O(5)#3	91.1(4)		

Symmetry transformations used to generate equivalent atoms:

#1 x-1/2,-y+3/4,-z+1/4 #2 -x+1,y-1/4,z-1/4 #3 -x+1/4,y,-z+1/4

#4 x-1/4,y+1/4,-z+1/2 #5 -x+3/4,-y+1/4,z-1/2

#6 -x+1/2,-y+1/2,-z #7 x+1/2,y,z+1/2 #8 x,-y+1/4,-z+1/4

#9 -x+1/4,-y+1/4,z #10 x+1/2,-y+3/4,-z+1/4 #11 -x+1,y+1/4,z+1/4

#12 -x+3/4,-y+1/4,z+1/2 #13 x+1/4,y-1/4,-z+1/2

#14 x-1/2,y,z-1/2

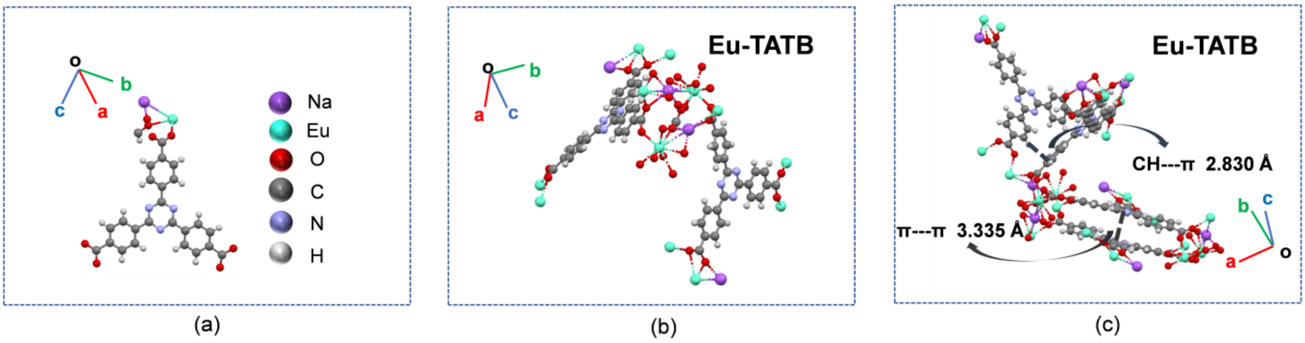

Figure S2. (a) Minimum asymmetric element structure of Eu-TATB. Deep purple = Na; lake blue = Eu; red = oxygen; gray = carbon; light purple = nitrogen; white = hydrogen. (b) Schematic diagram of Eu^{3+} coordination environment. (c) Eu-TATB crystals. CH-π, π-π short-contacts were shown in these stacked molecular conformations.

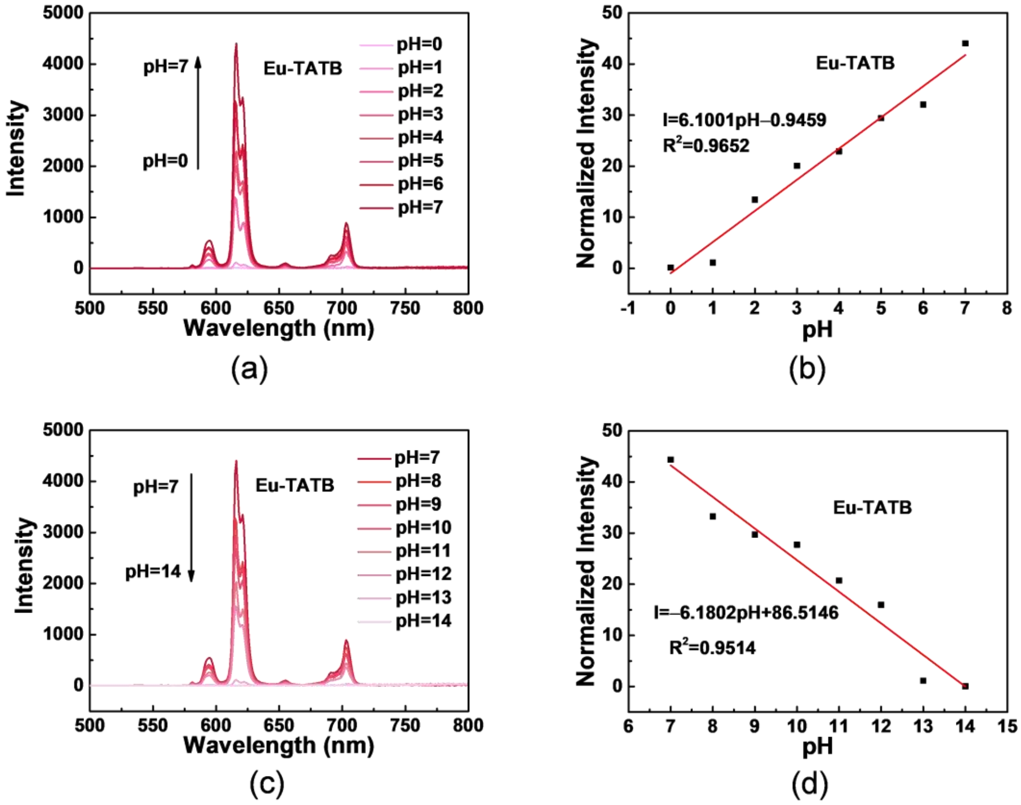

Figure S3. (a), (c) The change in fluorescence emission intensity of Eu-TATB treated by HCl/NaOH aqueous solutions with pH ranging from 0 to 14. (b), (d) The linear variation of the emission (614 nm) intensity for Eu-TATB treated by HCl/NaOH aqueous solutions with pH ranging from 0 to 14.

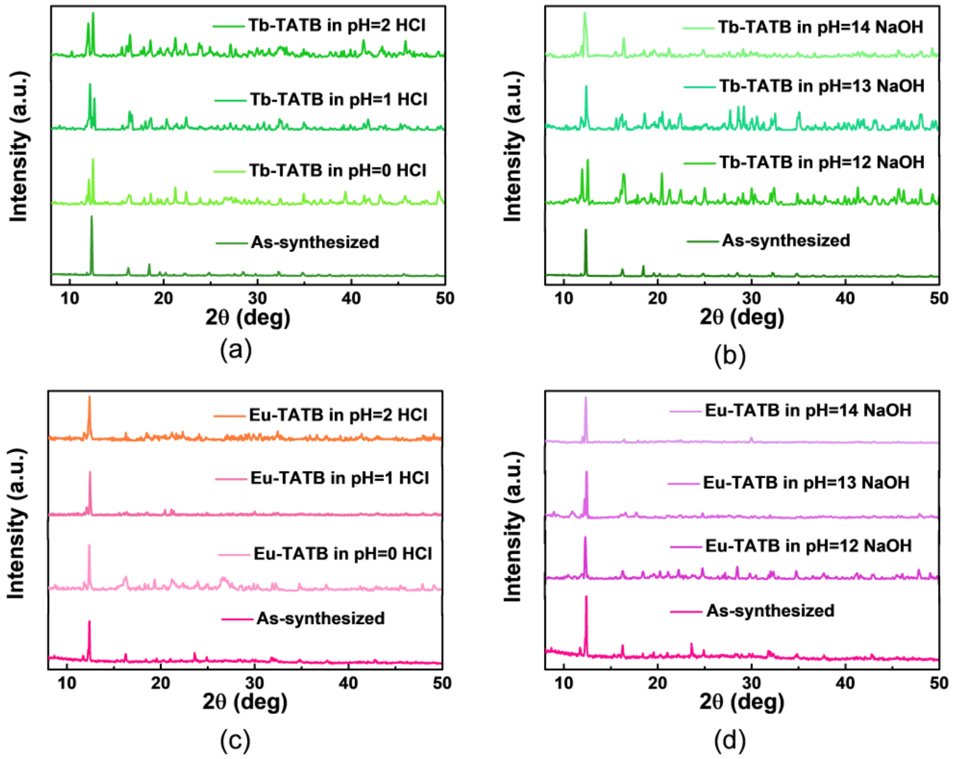

Figure S4. (a), (b) PXRD patterns of Tb-TATB after soaked in strong acid and base solutions for 12 hours. (c), (d) PXRD patterns of Eu-TATB after soaked in strong acid and base solutions for 12 hours.

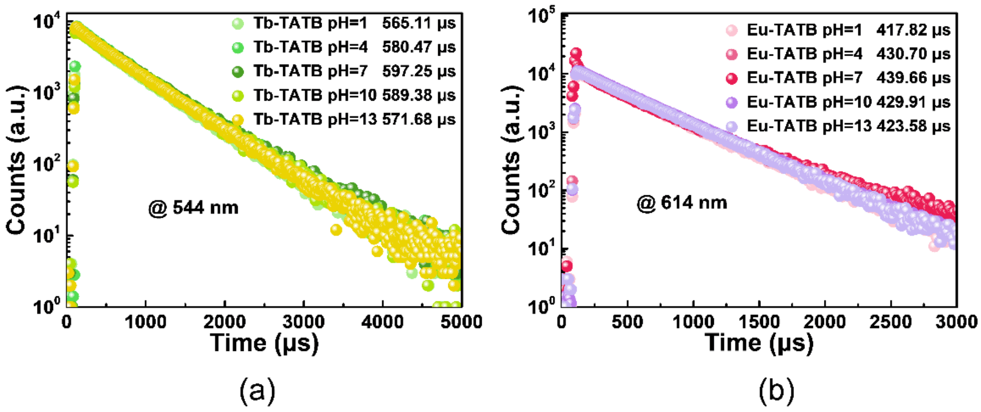

Figure S5. Phosphorescence decay curves of (a)Tb-TATB (λ_{em}=544 nm) and (b)Eu-TATB (λ_{em}=614 nm) after soaking in different pH values.

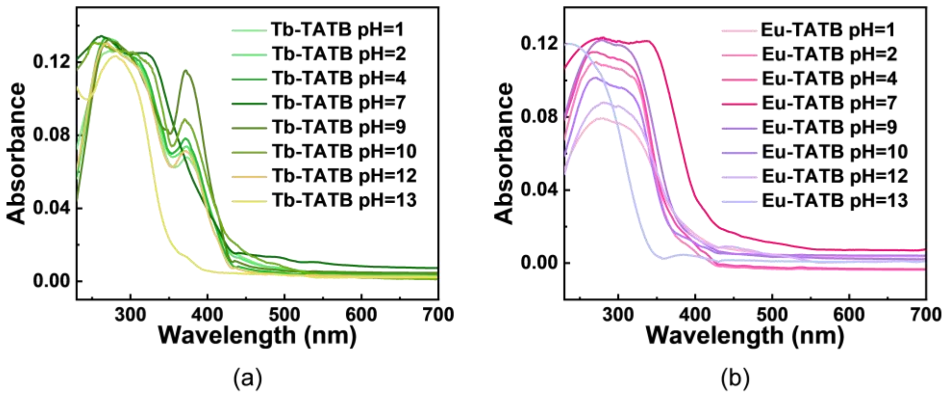

Figure S6. Solid-state UV-vis absorption spectrum of (a) Tb-TATB and (b) Eu-TATB in different pH.

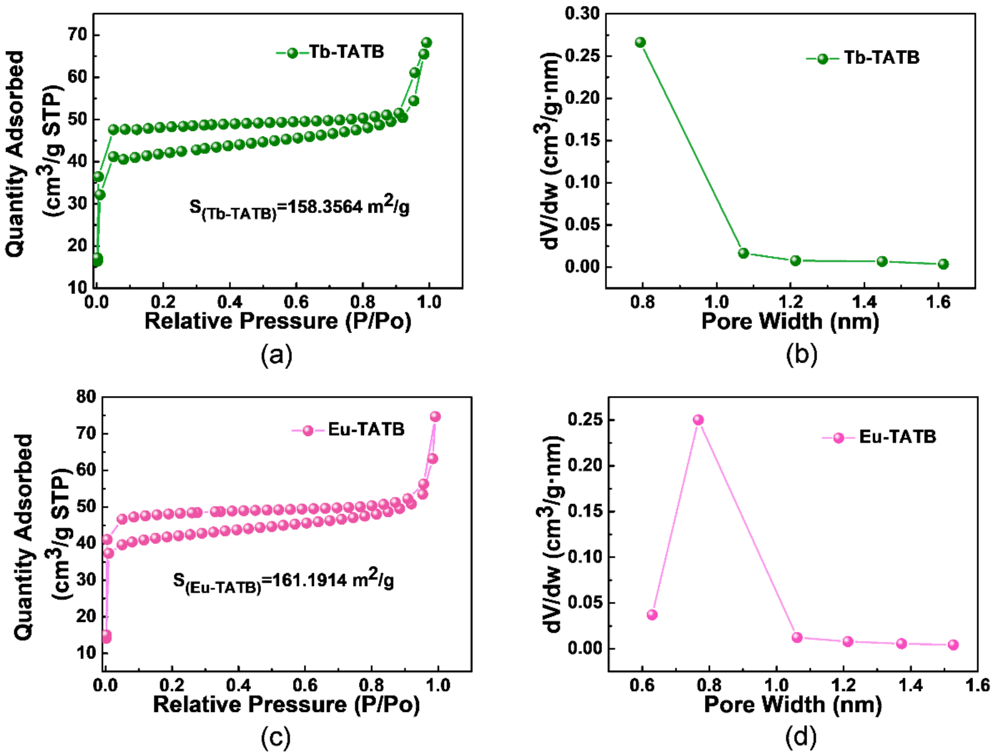

Figure S7. (a) Specific surface area and (b) micropore size distribution of Tb-TATB. (c) Specific surface area and (d) micropore size distribution of Eu-TATB.
